# Towards the Integration of an Islet-Based Biosensor in Closed-Loop Therapies for Patients With Type 1 Diabetes

**DOI:** 10.3389/fendo.2022.795225

**Published:** 2022-04-22

**Authors:** Loïc Olçomendy, Louis Cassany, Antoine Pirog, Roberto Franco, Emilie Puginier, Manon Jaffredo, David Gucik-Derigny, Héctor Ríos, Alejandra Ferreira de Loza, Julien Gaitan, Matthieu Raoux, Yannick Bornat, Bogdan Catargi, Jochen Lang, David Henry, Sylvie Renaud, Jérôme Cieslak

**Affiliations:** ^1^ Univ. Bordeaux, CNRS, Bordeaux INP, IMS, UMR 5218, Talence, France; ^2^ Tecnológico Nacional de México/I.T. La Laguna, Torreón, Mexico; ^3^ Univ. Bordeaux, CNRS, CBMN, UMR 5248, Pessac, France; ^4^ Cátedras CONACYT, Ciudad de México, Mexico; ^5^ Instituto Politécnico Nacional-CITEDI, Tijuana, Mexico; ^6^ Bordeaux Hospitals, Endocrinology and Metabolic Diseases Unit, Bordeaux, France

**Keywords:** type 1 diabetes, artificial pancreas, closed-loop simulation, insulin therapy, pancreatic islets, micro-electrode array, biosensor

## Abstract

In diabetes mellitus (DM) treatment, Continuous Glucose Monitoring (CGM) linked with insulin delivery becomes the main strategy to improve therapeutic outcomes and quality of patients’ lives. However, Blood Glucose (BG) regulation with CGM is still hampered by limitations of algorithms and glucose sensors. Regarding sensor technology, current electrochemical glucose sensors do not capture the full spectrum of other physiological signals, *i.e*., lipids, amino acids or hormones, relaying the general body status. Regarding algorithms, variability between and within patients remains the main challenge for optimal BG regulation in closed-loop therapies. This work highlights the simulation benefits to test new sensing and control paradigms which address the previous shortcomings for Type 1 Diabetes (T1D) closed-loop therapies. The UVA/Padova T1DM Simulator is the core element here, which is a computer model of the human metabolic system based on glucose-insulin dynamics in T1D patients. That simulator is approved by the US Food and Drug Administration (FDA) as an alternative for pre-clinical testing of new devices and closed-loop algorithms. To overcome the limitation of standard glucose sensors, the concept of an islet-based biosensor, which could integrate multiple physiological signals through electrical activity measurement, is assessed here in a closed-loop insulin therapy. This investigation has been addressed by an interdisciplinary consortium, from endocrinology to biology, electrophysiology, bio-electronics and control theory. In parallel to the development of an islet-based closed-loop, it also investigates the benefits of robust control theory against the natural variability within a patient population. Using 4 meal scenarios, numerous simulation campaigns were conducted. The analysis of their results then introduces a discussion on the potential benefits of an Artificial Pancreas (AP) system associating the islet-based biosensor with robust algorithms.

## Introduction

**Figure 1:**
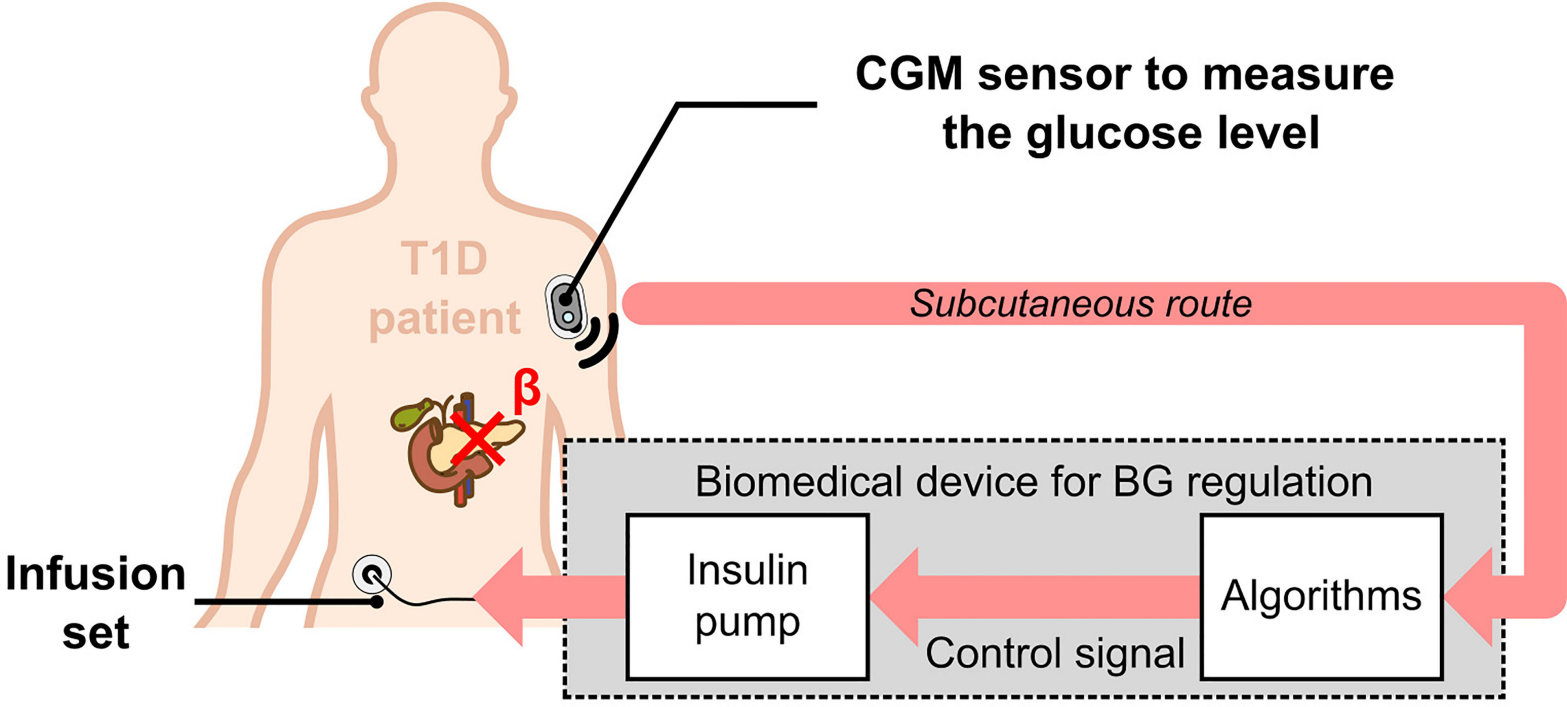
Principle of the Artificial Pancreas for T1D treatment. An electrochemical CGM sensor continuously measures subcutaneous glucose concentrations, which reflect blood glucose concentration. This information is then processed by algorithms (controller, bolus calculator, alarms …), connected to an insulin pump to deliver the appropriate amounts of insulin.

Destruction of pancreatic *β*-cells leads to absolute insulin deficiency in Type 1 Diabetes (T1D) and concerns 5 to 10% of the estimated 463 million cases of diabetes worldwide in 2019, expected to rise to 700 million by 2045 according to the International Diabetes Federation (1). In this context, the development of Artificial Pancreas (AP) systems, composed of a Continuous Glucose Monitoring (CGM) sensor fitted with a pump to deliver insulin, is becoming the standard for T1D treatment (2, 3). CGM relies on subcutaneous glucose measurement *via* electrochemical electrodes and algorithms are used to control the pump and safely manage the insulin delivery (**Figure 1**).

In spite of improvements relative to hypoglycaemia prevention (4) and hyperglycaemia mitigation (5, 6), Blood Glucose (BG) regulation with the AP is still biased by the limitations of algorithms (7) and technologies used in commercial glucose sensors (8). Current electrochemical approaches in glucose sensors do not consider the whole spectrum of nutrients and do not respond to all physiological situations (e.g., contribution of intestinal hormones to insulin secretion after a meal, physical activity, stress), which all modulate insulin requirements. Regarding algorithms, variability between and within patients, also referred to as inter- and intrapatient variability, remains the main challenge for optimal glycaemia regulation with closed-loop therapies. As a consequence, only partially automated closed-loop systems are currently accepted for therapy in the US and Europe, *i.e*., the T1D patient still has to announce meals and calculates carbohydrate intake to command himself the bolus insulin injections (3). Alleviating some of these issues, specifically in the case of unstable diabetes, would lower the barriers to closed-loop therapy for patients, with a mitigation of patient’s workload and anxiety.

**Figure 2:**
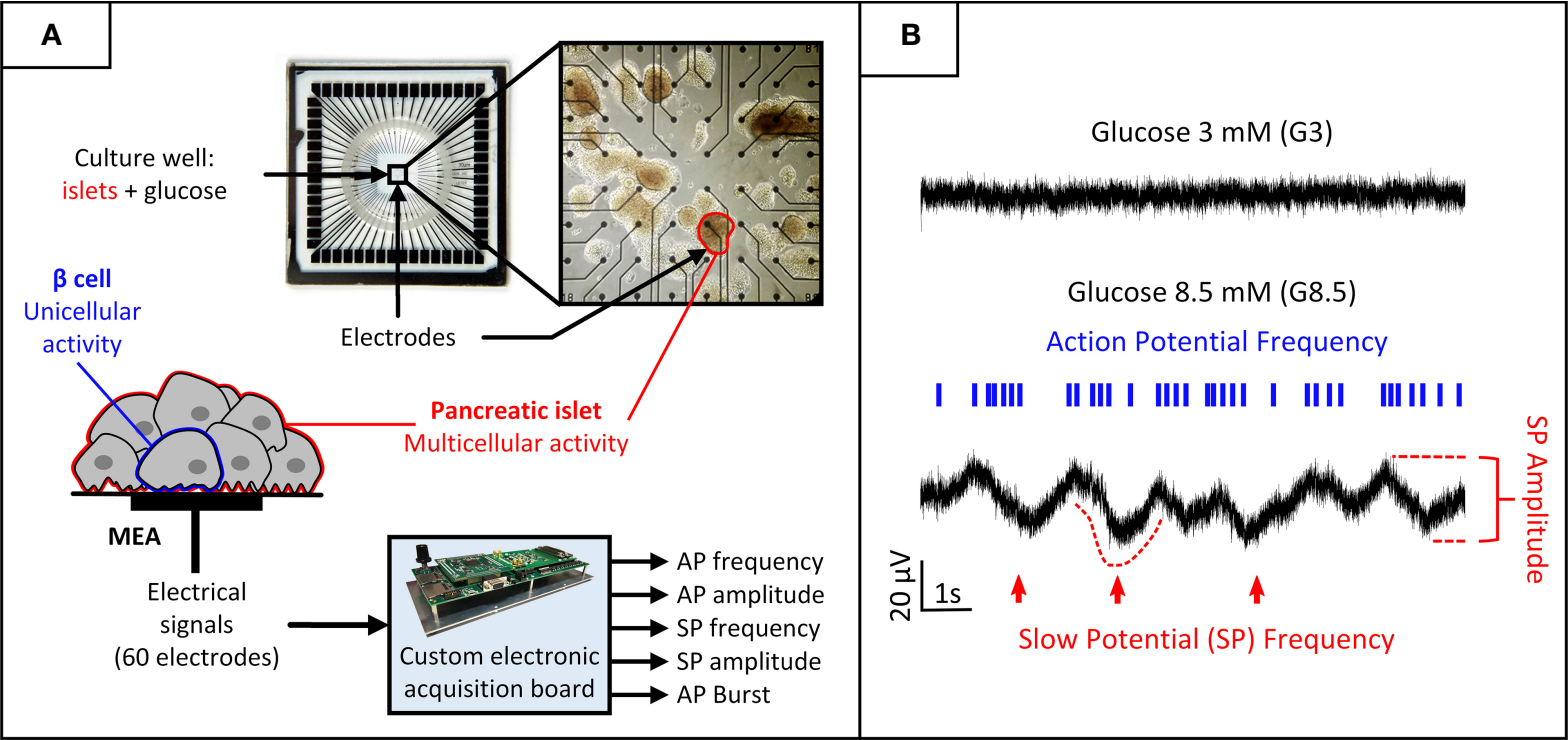
Biosensor principle: acquisition and processing of electrical biosignals generated by pancreatic islets cultured on MEAs and stimulated by increasing glucose levels. **(A)** Pancreatic islets cultured on MEAs. Glucose can be introduced in the culture chamber to stimulate the cells. Each electrode in the MEA captures a combination of uni- and multicellular activity generated by the neighbouring islets. A custom electronic board performs online digital signal processing on the recorded biosignals to extract features of interest for each electrode. **(B)** The electrical activity is modulated by glucose concentration. Low glucose inhibits activity and high glucose induces two signals of interest generated by β-cells, representative of uni- and multicellular activity: action potentials and SPs. Action potentials are mainly characterized by their frequency and organisation in bursts, and SPs by both their frequency and amplitude. [From (13)].

To overcome the shortcomings of enzymatic sensors, our initiative aimed at developing a biosensor which integrates a Micro Electrode Array (MEA) containing a few murine or human islets linked to real-time/online signal processing (9–12). Pancreatic islets are the “in-born” sensors and actuators, optimally shaped by evolution, to ensure regulation of glucose homeostasis under various natural circumstances and lifestyles. The goal is to design a sensor capable of “seeing” the whole-body physiological interactions, as opposed to the classical glucose-only sensors. Islets, composed of several (hundreds of) excitable cells, display continuous oscillations, reflecting its orchestrated behaviour. Action potentials and slow oscillations – named Slow Potentials (SP) - can be recorded extracellularly using MEAs (**Figure 2**). Islets SPs have amplitudes in the range of few tens of microvolts, frequency components ranging between 0.2 and 2 Hz (11), and their characteristics are closely correlated to insulin secretion dynamics (14). Signal treatment raises challenges when processing it online and in real-time for *in vivo* applications. Decoding information from the recorded signals requires analogue pre-processing by amplifiers and filters, followed by digital processing with statistical, frequency, or temporal analysis to perform feature extraction and produce relevant metrics (15). Furthermore, adaptive decoding is essential to take into account variations in signal and electrode properties, particularly for chronic recordings (16). This sensor technology has been patented in 2013 (17).

Building on promising results of the previously developed and patented glucose bio-device, which integrates multiple physiological signal information (17, 18), a consortium has been created in 2019 to assess the possibility to integrate this islet-based biosensor in closed-loop therapies for patients with T1D. This consortium started the collaboration in a national project named DIABLO, supported by the French National Agency for Research (ANR). Preliminary work (19) provided guidelines for the controller tuning with an *in silico* methodology based on clinically-relevant criterion: a meta-heuristic method (genetic algorithm (GA)-based optimization technique) is used with the BG risk index (20). The core element of the GA-based protocol is the UVA/Padova T1DM Simulator (T1DMS - v3.2) (21). This computer model of the human metabolic system simulates the glucose-insulin dynamics in T1D patients, and is approved by the US Food and Drug Administration (FDA) as an alternative for pre-clinical testing of insulin therapies, including closed-loop algorithms (22). Using the T1D adult cohort of the simulator, a first comparison between two AP systems (a biosensor-based one and a CGM-based one) was presented in (13). Thanks to individualised controller parameters, satisfactory performance was achieved with the biosensor-based AP system, even with a simple proportional-derivative controller associated to continuous basal infusion (PD_BASAL_). This regulation scheme was as efficient as standard treatments with unannounced meals (no bolus strategy was implemented).

Another objective of the DIABLO project lies in the use of control theory to tackle the variability observed between and within patients in a real T1D population. For that purpose, it is necessary to have a relevant model capable of accurately capturing glucose-insulin dynamics. This topic has received a great attention in the last decade, with different type of models: from Linear Time Invariant (LTI) (23, 24) to Linear Parameter Varying (LPV) ones (25–27). In the DIABLO project, it has been proposed to derive a family of LTI models of thirteen-order from the UVA/Padova simulator to capture the dynamics from the subcutaneous insulin to the subcutaneous glucose in T1D patients. This set of LTI models is composed of a nominal LTI model fitted with an uncertainty block and it can be used for design and analysis purpose. Based on this modelling, a unique and robust Proportional-Integral-Derivative (PID) has been designed for the T1DMS adult cohort in (28). Results reported in (28) showed that BG regulation fitted with a basic bolus strategy of 2 units of insulin applied during the meal announcement, provided quite similar performances with respect to the individualised PID controllers of (29). These results motivated the use of a unique and robust controller to generate a continuous basal insulin injection. With the current technology of CGM sensors, it appears, however, necessary to couple this basal delivery with a bolus insulin injection protocol to improve the time in the so-called normo-glycaemic range (70mg/dl < BG < 180mg/dl) for counteracting the meal intakes. From (3, 30), this strategy has been adopted by the current commercial AP systems on the market like MiniMed 780G (CE and FDA approval), Diabeloop (CE approval), Tandem t:slim X2 (FDA approval, CE approval in progress) and Omnipod Horizon (FDA approval in progress) where the bolus strategy involves assistance from the T1D patient, *i.e*. the patient has to calculate carbohydrate intake to precisely dose insulin boluses.

In the present work, we intend to highlight the benefits of numerical simulation (with the UVA/Padova T1DMS) to address this issue and establish *in silico* proofs of concept for the DIABLO project. In particular, we propose a method to define meal scenarios based on patients’ body weight to better account for the interpatient variability in energy requirements and define more realistic meal scenarios. These scenarios are then used to assess the two different closed-loop solutions we already mentioned: the first one uses a GA-based controller tuning method (13, 19) and the other one based on a robust control theory approach (28, 31). With this second approach, we also propose a meal size-independent bolus strategy, slightly individualised by integrating the Carbohydrate-to-Insulin Ratio (CIR) in the bolus calculator rule. The objective here is to alleviate patient’s workload and anxiety, while keeping him involved in the therapy management, *i.e*., the patient still has to announce sizeable glucose intakes (meals). From an analysis of the *in silico* results, we will finally discuss the proposal of an original AP paradigm where the dissimilarity between a commercial CGM sensor and our biosensor could be used advantageously, to better handle inter- and intrapatient variability in diabetes treatment and care.

## Materials and Methods

### UVA/Padova Simulator

Simulators of human metabolic system based on the glucose-insulin dynamics, have been shown to be useful in developing diabetes treatment solutions (32). Such testing environments give the opportunity to assess the performance of algorithms with costs and time savings, and avoid ethical questions. In particular, the UVA/Padova T1DMS is the only simulation tool, approved by the US Food and Drug Administration (FDA), as an alternative for pre-clinical testing of closed-loop algorithms (22, 33). T1DMS includes mathematical models of glucose-insulin dynamics, and several types of CGM sensors with realistic imperfections on the glucose measurement, insulin pumps and a simulation block dedicated to algorithm assessment. We used here the latest commercial version (v3.2) based on the equations given in Dalla Man et al. (21). This version includes a cohort of 33 T1D patients (11 adults, 11 adolescents, and 11 children). Hence, it is possible to simulate the effect of realistic meal scenarios on various virtual patients treated with the proposed closed-loop insulin solutions. However, it has to be noted that the considered version (v3.2) of the T1DMS involves the following working assumption:

*Assumption 1:* The glucose-insulin dynamics are not modulated by the circadian variability of insulin sensitivity.

The authors are aware that such assumption can limit the significance of multi-meal simulations. This choice has been made to not question or alter the human metabolic model approved by the FDA. A deeper analysis of this topic will be given in the discussion section.

### A Meta-Heuristic Method to Design an Islet-Based Closed-Loop Therapy

In real T1D populations, a large inter-patient variability is observed in terms of sensitivity to insulin, body weight, and T1D duration. This variability is a serious issue in designing easily adjustable AP systems as the amount of insulin required to mitigate postprandial hyperglycaemia greatly varies among patients. To account for this variability as well as to ensure reliability and stability of the closed-loop system, a fine tuning of the AP controller’s parameters is necessary.

#### Controller Tuning

**Figure 3:**
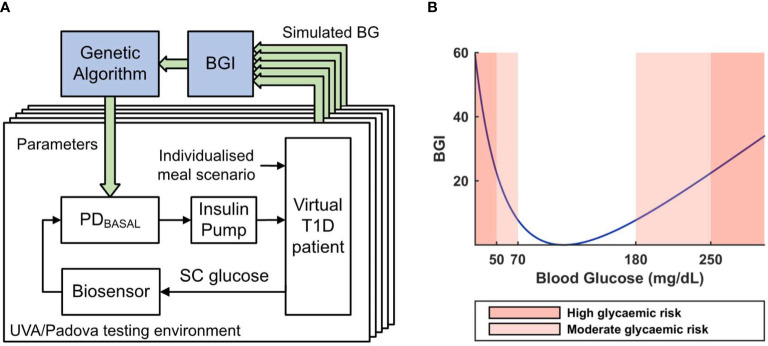
**(A)** Working principle of the Genetic Algorithm-based controller tuning method. 5 single meal scenarios are simulated for various controller parameter combinations. The closed-loop performance of each combination (averaged on the 5 scenarios) is assessed with a BGI-based cost function to iteratively tune the parameters of a PD_BASAL_ controller. SC glucose denotes the subcutaneous glucose concentration. **(B)** Blood Glucose Index (BGI) risk unction plot.

In the first part of this work, a GA^1^-based optimization technique is used to tune a PD_BASAL_ controller for each adult patient of the T1DMS cohort with respect to a clinically-relevant objective metric: the Blood Glucose Index (BGI) (**Figure 3**). This metric is a known indicator of the clinical risk associated with a given blood glucose level (20). The BGI risk function is defined as follows:


(1) $$\text{BGI}(G)=10\times {\left(1.509\times (ln{\left(G\right)}^{1.084}-5.381)\right)}^{2}$$

where *G* is the glucose level measured in mg/dl. By minimizing the mean BGI over a series of single meal scenarios, our GA-based algorithm can find controller parameters, which minimizes the clinical risk associated with the closed-loop regulation of the patient’s glycaemia.

#### Controller Design

As a first step, this method was applied to the tuning of simple Proportional-Integral-Derivative (PID) controllers to handle the diffusion delays induced by subcutaneous glucose measurement and insulin infusion. Prior to (19), many variants of the traditional PID architecture were tested and a PD architecture associated with a subject specific basal infusion of insulin (PD_BASAL_) was finally selected. This controller architecture provided good performance and allowed us to reduce the number of parameters to tune to 2, thus increasing the GA convergence speed. The corresponding discrete-time controller is represented by:


(2) $$C(z)=K_{p}\left(1+\frac{T_{d}}{T_{s}}\times \frac{z-1}{z}\right)$$

where *T_s_* = 5 min is the sampling period of the PD_BASAL_ controller, *T_d_* its derivative time constant, and *K_p_* its proportional gain. A constant patient-specific basal insulin infusion rate provided by the T1DMS for each patient, is then summed to the controller output. More details about the islet-based sensor and its integration in a BG regulation closed-loop are given in (13); more details about the controller tuning methodology are given in (13, 19).

#### Body Weight-Dependent Meal Scenario Definition

The tuning method presented above has already proven its efficiency to individually tune the controller parameters of a CGM-AP for the 11 virtual T1D adults of the T1DMS patient cohort (19). The method was then refined to better handle the CGM sensor noise and applied to the tuning of our biosensor-based AP (Bios-AP) controller  (13).

**Figure 4:**
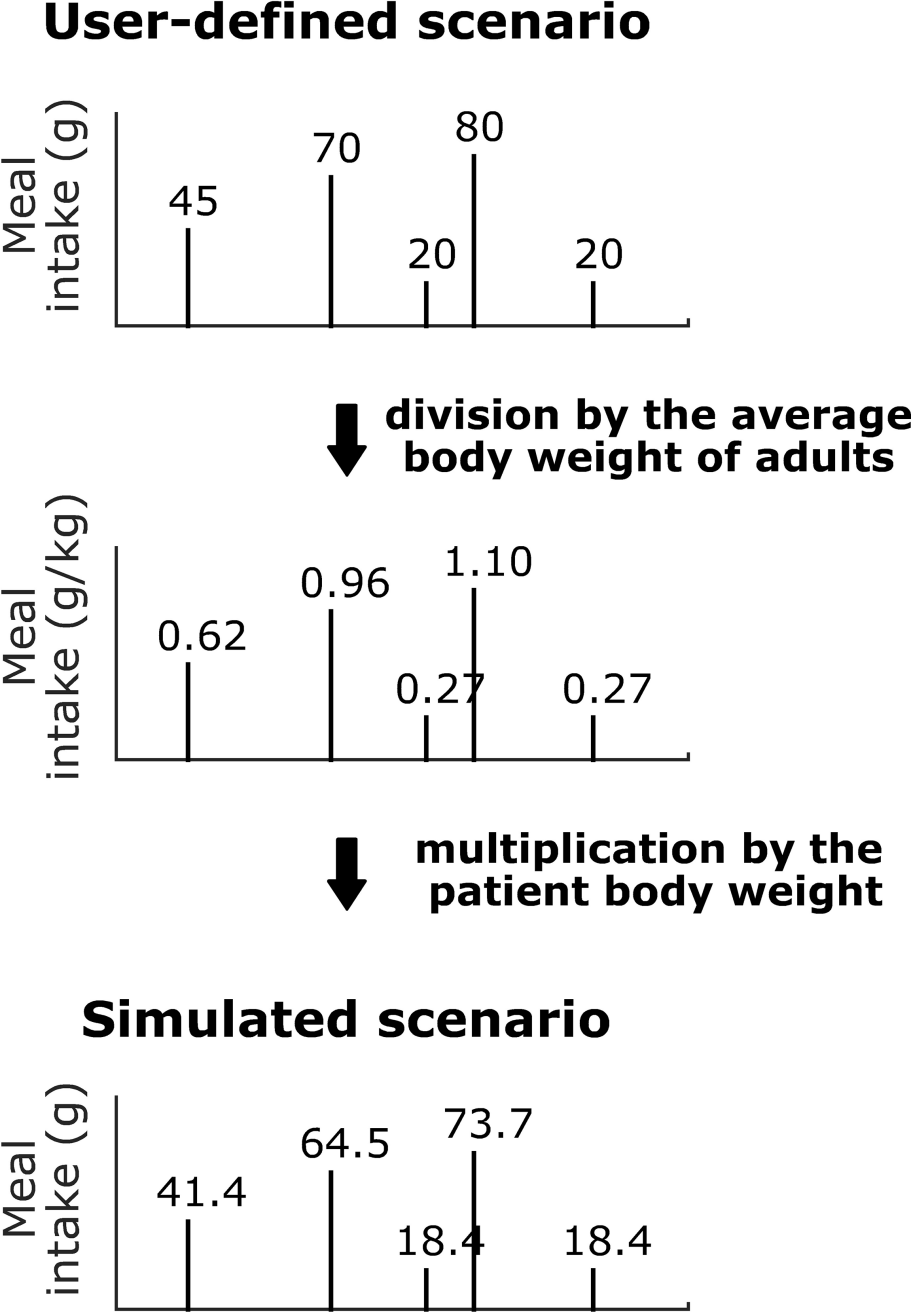
Body weight-dependent definition of the glucose intake scenarios. Example for adult#005 of the T1DMS.

Real T1D patients have specific energy needs related to their individual metabolisms, ages, sexes and lifestyles. Using a unique meal scenario to evaluate the performance of closed-loop systems on a T1D population (either *in vivo* or *in silico*) therefore seems inadequate as most of the patients would receive either an under- or overstimulation by the unique meal scenario relative to their specific needs. To address this issue, and thus better account for the interpatient variability of energy requirements, we propose here a method to individualise the meal scenarios. To keep it simple, individualisation was performed using a single parameter. Among the patient’s parameters provided by the T1DMS we chose the body-weight as it is the parameter which best represents patient’s singularity, i.e., age, sex, metabolism, and lifestyle. To achieve meal scenario individualisation, each glucose intake of the user-defined scenario is divided by the average body weight of the 11 adults to obtain a meal scenario whose glucose intakes are defined in grams of glucose per kilogram (of body weight). The individualised scenarios which are actually simulated are then generated proportionally to each patient’s body weight, see **Figure 4**. This method is implemented as a MATLAB function which seamlessly integrates the simulator execution flow. The function reads the user-defined meal scenario and generates individualised scenarios, while ensuring that the average daily glucose intake computed on all generated scenarios is the daily glucose intake of the user-defined scenario. In our previous work (13), validation scenarios were designed to match the daily glucose intake reported in the literature for American T1D adults (235 grams of glucose in average) (34). The main advantage of this method is that it maintains, by design, this realism as the scenario defined by the user serves to set the average scenario on the whole adult population. Note that such “normalization” to the body weight is also commonly used for animal *in vivo* glucose tolerance tests to avoid inter-individual bias (35).

Using the GA-method presented in (13, 19) with individualised scenarios, a new set of controller parameters was generated for the Bios-AP, and for the 10 virtual T1D patients of the T1DMS (the 11^th^ adult#average patient was not used in this study).

### Simulation Benefits for Robust Control Problem Formulation

**Figure 5:**
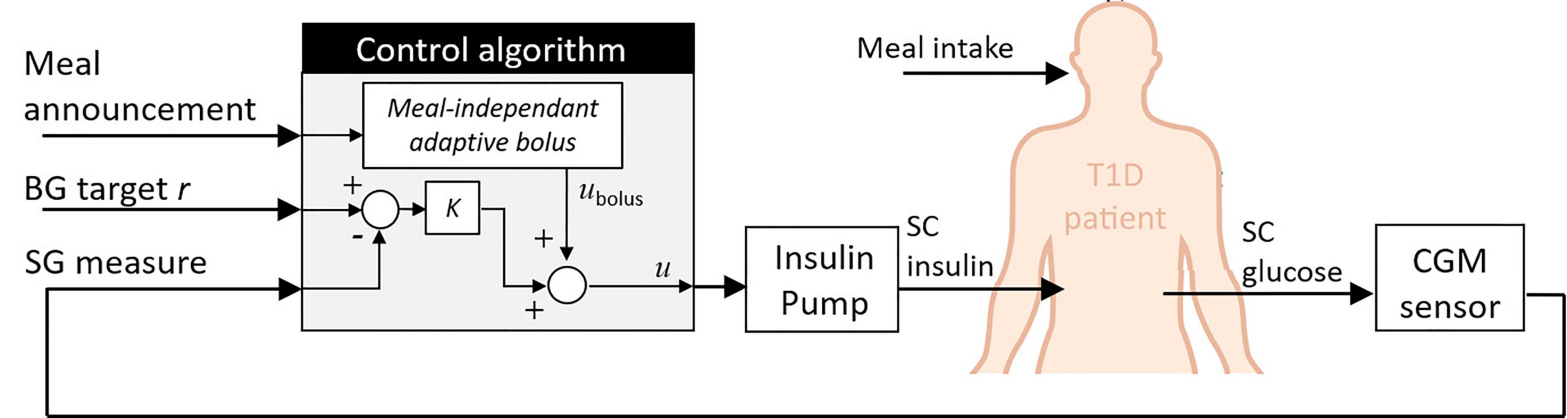
Standard setup for closed-loop insulin therapy. In this work, the controller *K* is a unique robust controller for the whole T1D patient population. Personalization is deported in the bolus calculator rule.

In parallel to the development of an islet-based closed-loop architecture for BG regulation, we also attempted to formulate a control problem compliant with a robust solution. For that purpose, it has been proposed to derive a family of LTI models of thirteen-order from the UVA/Padova simulator able to capture the interpatient variability in the glucose-insulin dynamics. These models were then used to design a unique feedback controller *K*(*s*), for a population of T1D patients, which delivers a control signal called insulin basal. To quickly react to food intake, a meal announcement feature was implemented in this second part to trigger the delivery of meal boluses. Contrary to more standard meal bolus features involving a patient-provided estimation of the quantity of ingested carbohydrates (36), we developed a bolus strategy which diminish patient’s workload and anxiety by only requesting a meal announcement, *i.e.*, a constant insulin bolus is delivered for each sizeable meal (breakfast, lunch and dinner). This meal-independent bolus feature was individualised by integrating the patient CIR^2^ knowledge of the clinician in charge of the T1D patient. The control algorithm, proposed in this subsection, thus delivers the following insulin signal *u*(*t*):


(3) $$u(t)=\{\begin{array}{cc} K(r(t)-SG(t))+u_{\text{bolus}} & \text{for one minute at meal announcement}\\ K(r(t)-SG(t)) & \text{otherwise} \end{array}$$

where *SG*(*t*) is the subcutaneous glucose signal delivered by a CGM sensor. *r*(*t*) is the glucose target. With a duration of one minute after a sizeable meal announcement, the signal *u*_bolus_ is given by the following mathematical expression:


(4) $$u_{\text{bolus}}=12000L(\text{CIR}),\;L(\text{CIR})=\{\begin{array}{c} 1\text{ if CIR}>15\text{g}/\text{U}\\ 2\text{ if CIR}\leq 15\text{g}/\text{U} \end{array}$$

where the value of 12000 pmol/min (2 unities of fast insulin) has been chosen to be compliant with the requirements of (29). This magnitude can be adapted by considering the CIR of the T1D patient to schedule the adaptive gain *L*, see equation (4). Hence, the retained closed-loop insulin setup in this sub-part obeys to the architecture shown in **Figure 5**.

In the following subsection, we first provide guidelines showing how it is possible to derive a family of linear models for the considered population of T1D patients. Next, a robust control technique was used for control design purpose.

#### Getting a Family of Linear Models of T1D Patient Population

From (21), the nonlinear dynamical model of a T1D patient can be written according to:


(5) $$\{\begin{array}{l} \dot{x}(t)=f(x(t),u(t),\theta (t))\\ y(t)=C_{0}x(t) \end{array}$$

where $C_{0}=(0_{1\times 12}V_{G}^{-1}\;0_{1\times 5})$ with *x*(*t*) ∈ ℝ^18^, *u*(*t*) ∈ ℝ^3^, θ(*t*) ∈ ℝ^9^ and *y*(*t*) ∈ ℝ are respectively the model state, input, time-varying parameter and output vectors, with the functional *f*: ℝ^18^ × ℝ^3^ × ℝ^9^ →ℝ^18^. All time-varying parameters and the physiological parameter *V_G_* are defined in (21). As we were considering closed-loop insulin systems, the model given by equation (5) was reduced to a state vector of 13 states by taking out the contribution of the last 5 states (*x*_14_, *x*_15_,…, *x*_18_) relative to the glucagon dynamics. Hence, the reduced model became


(6) $$\{\begin{array}{l} {\dot{x}}_{r}(t)=f_{r}(x_{r}(t),u_{r}(t),\theta_{r}(t))\\ y(t)=Cx_{r}(t) \end{array}$$

where $C=(0_{1\times 12}V_{G}^{-1})$ with *x_r_* (*t*) ∈ ℝ^13^, *u_r_* (*t*) ∈ ℝ^2^, θ*_r_* (*t*) ∈ ℝ^7^ and *f_r_*: ℝ^13^ × ℝ^2^ × ℝ^7^ →ℝ^13^. *u_r_* (*t*) = (*u*_1_(*t*) *u*_2_(t))*^T^* where *u*_1_ refers to the carbohydrate intake (*i.e*. the meal) and *u*_2_ corresponds to the insulin infusion rate, which is delivered to the patient through an insulin pump.

To obtain a family of linear models able to fit the nonlinear model equation (6), the set of operating points $\left(x_{r}^{*},u_{r}^{*}\right)$ had to be choosen judiciously, *i.e*., the time-varying parameters *θ_r_* were constant on a time interval described later. For each operating point $\left(x_{r}^{*},u_{r}^{*}\right)$ , a first-order Taylor approximation was thus performed and the nonlinear model equation (6) were reformulated as follows:


(7) $$\{\begin{array}{l} \delta {\dot{x}}_{r}(t)=A(x_{r}^{*},u_{r}^{*})\;\delta x_{r}(t)+B(x_{r}^{*},u_{r}^{*})\;\delta u_{r}(t)\\ \delta y(t)=C\delta x_{r}(t) \end{array}$$

where $\delta x_{r}(t)=x_{r}(t)-x_{r}^{*}$ and $\delta u_{r}(t)=u_{r}(t)-u_{r}^{*}$ . δ*y*(*t*) is the variation of the output with respect to the fasting basal glucose *G_b_* and *A*, *B* are the Jacobian matrices of vector field *f_r_* with respect to *x_r_* and *u_r_*, evaluated at $\left(x_{r}^{*},u_{r}^{*}\right)$ . The key element was then to define a set of values $\left(x_{r}^{*},u_{r}^{*}\right)$ sufficiently dense to obtain an accurate approximation of equation (6) with (7). To proceed, the nonlinear model of the T1DMS was used to simulate a single meal scenario with basal insulin input (*u*_2_ = *I_b_*). The meal corresponded to 50 g of carbohydrates, ingested during 15 min (i.e. *u*_1_ = 3333 mg/min). The basal insulin *I_b_* is the proper quantity of insulin that allows to reach a steady-state condition during fasting periods (37). For the considered population of adult T1D patients, we have 94.6 pmol/min ≤ *I_b_* ≤ 150.0 pmol/min. From this simulation, the spatial discretization is achieved on *x_r_* and *u_r_* in order to produce a set of adequate values for $\left(x_{r}^{*},u_{r}^{*}\right)$ such that time-varying parameters *θ_r_* are constant on the considered interval, *i.e*. let the simulation time horizon [0, *T*] be divided into subintervals as follows: 0 = *t*_0_ < *t*_1_ < ··· *t_n_* = *T*. The set λ = (λ_0_,λ_1_,…, λ*_k_*,λ*_n_*) is defined such as:


(8) $$\{\begin{array}{l} \lambda_{0}\in \left[t_{0},\frac{t_{1}}{2}\right]\\ \lambda_{k}\in \left[\frac{t_{k}+t_{k-1}}{2},\frac{t_{k+1}+t_{k}}{2}\right]\;\\ \lambda_{n}\in \left[\frac{t_{n}+t_{n-1}}{2},t_{n}\right] \end{array}\text{for}\;1\leq k<n$$

**Figure 6:**
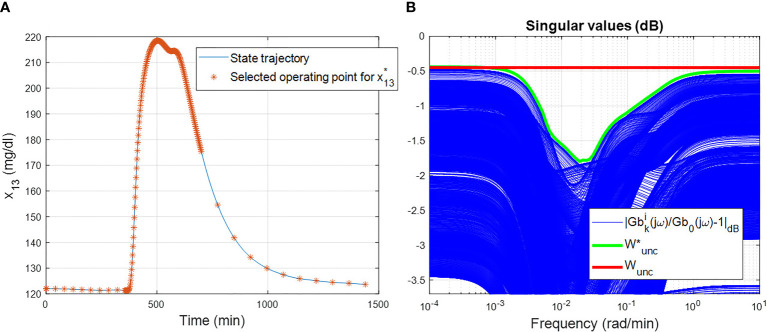
Use of the T1DMS for modelling purpose: two important steps **(A)** Spatial discretization for the considered single meal scenario on the 13th states of the nonlinear model in UVA/Padova simulator for the patient adult#001 of T1DMS. **(B)** Results of the constructive solution to obtain the upper LFT of the entire family of linear models. In blue, it is the frequency behaviour of $\left|G_{k}^{i}(j\omega )/G_{0}(j\omega )-1\right|\forall i,k,\omega$ . The optimal solution $W_{\text{unc}}^{*}$ is plotted in green and the retained value *W*_unc_ for this study case is plotted in red.

On each subinterval, *t* ∈ λ*_k_* for *k* ∈{0,…, *n*}, a linear model for each patient denoted with patient index *i* = {1,…,11} is deduced as


(9) $$\{\begin{array}{l} \delta {\dot{x}}_{r}^{i}(t)=A_{k}^{i}\;\delta x_{r}^{i}(t)+B_{k}^{i}\;\delta u_{r}^{i}(t)\\ \delta y^{i}(t)=C_{k}^{i}\;\delta x_{r}^{i}(t) \end{array}$$

where $B_{k}^{i}=(B_{1_{k}}^{i}B_{2_{k}}^{i})$ with $B_{1_{k}}^{i}B_{2_{k}}^{i}\in {\text{R}}^{13\times 1}$ . **Figure 6A** shows the spatial discretization for the considered scenario. This protocol was thus repeated several times (a total of *n* = 222 models per patient) in order to have a family of linear models able to guarantee a good approximation of the nonlinear model equation (6), see (28) for more details.

Before formulating the control design problem, two sources of uncertainty must be considered: *i*) the inter- and intra-patient variability within a T1D population due to patient’s characteristics (*e.g*., fasting basal, total daily insulin need, weight) and *ii*) the dynamics of the glucose diffusion from the intravascular space to the subcutaneous one. Note that the output of model equation (9) gives information on the 13^th^ model state corresponding to the level of subcutaneous glucose (*SG*(*t*)). To have information of BG level, it is necessary to refer to the 4^th^ model state.

Regarding the uncertainty of patient’s characteristics, the so-called unstructured multiplicative uncertainty form (38) is used to derive the family of linear models (Equation 9), which can be rewritten by using the Linear Fractional Transformation (LFT) representation according to:


(10) $$\begin{array}{c} Gb_{k}^{i}(s)={\underline{C}}_{k}^{i}{\left(sI-A_{k}^{i}\right)}^{-1}B_{2k}^{i}\;\forall i,k\\ =Gb_{0}(s)(1+W_{\text{unc}}(s)\Delta_{b}(s))={ℱ}_{u}(P_{b}(s),\Delta_{b}(s)) \end{array}$$

where the matrix ${\underline{C}}_{k}^{i}$ is used to refer to the 4^th^ state of equation (9). ℱ*_u_* is the upper LFT defined as ℱ*_u_* (*M*, *N*) = *M*_22_ + *M*_21_ N(*I* – *M*_11_ *N*)^–1^ *M*_12_. *W*_unc_(*s*) is a wheigting function used to normalize the uncertainty Δ*_b_*, ||Δ*_b_* ||_∞_≤1. Hence, *W*_unc_ has to guarantee:


(11) $$|W_{\text{unc}}(j\omega )|\;\geq \;\left|\frac{Gb_{k}^{i}(j\omega )}{Gb_{0}(j\omega )}-1\right|\;\forall i,k,\omega$$

Equation (11) gives a constructive solution to determine the couple (*Gb*_0_, *W*_unc_). To have the smallest conservative LFT, the optimal solution $\left(Gb_{0}^{*},W_{\text{unc}}^{*}\right)$ is constructed such that ||*W*_unc_||_∞_ is minimal. This optimization problem leads to the results given in **Figure 6B**, where $W_{\text{unc}}^{*}$ is found of order 11 to perfectly fit the upper bound. However, choosing a simple constant for *W*_unc_ ≈ 0.45 leads to a LFT ℱ*_u_* (*P_b_* (*s*), Δ*_b_* (*s*)), which is less complex (dimension of Δ*_b_* is one), with a small conservativeness since the maximum gap between the optimal solution $W_{\text{unc}}^{*}$ and *W*_unc_ is inferior to 1.5*dB*. Towards this end, the constant solution is retained in this study case.

Next, a parametric uncertainty is considered to integrate the time lag variability in T1D patients (between 6.83 and 10.83 min for the adult cohort of T1DMS) of glucose from intravascular to interstitial space (39). A deeper analysis of the equation (4) reveals that this variability is reflected by a gain variation of the transfer between the 4*th* (BG level) and the 13th (SG level) state. Such variation can be easily captured by an upper LFT so that


(12) Gsc(s)ki=Fu(PSC(s),ΔSC),∀i,kΔSC∈ℝ:||ΔSC||∞≤1

where Δ*_sc_* is the uncertainty block used to capture this variability. The input of Gsc(s)ki must be the BG level and its ouptut corresponds to the SG one.

#### Design of the Unique Controller *K*

**Figure 7:**
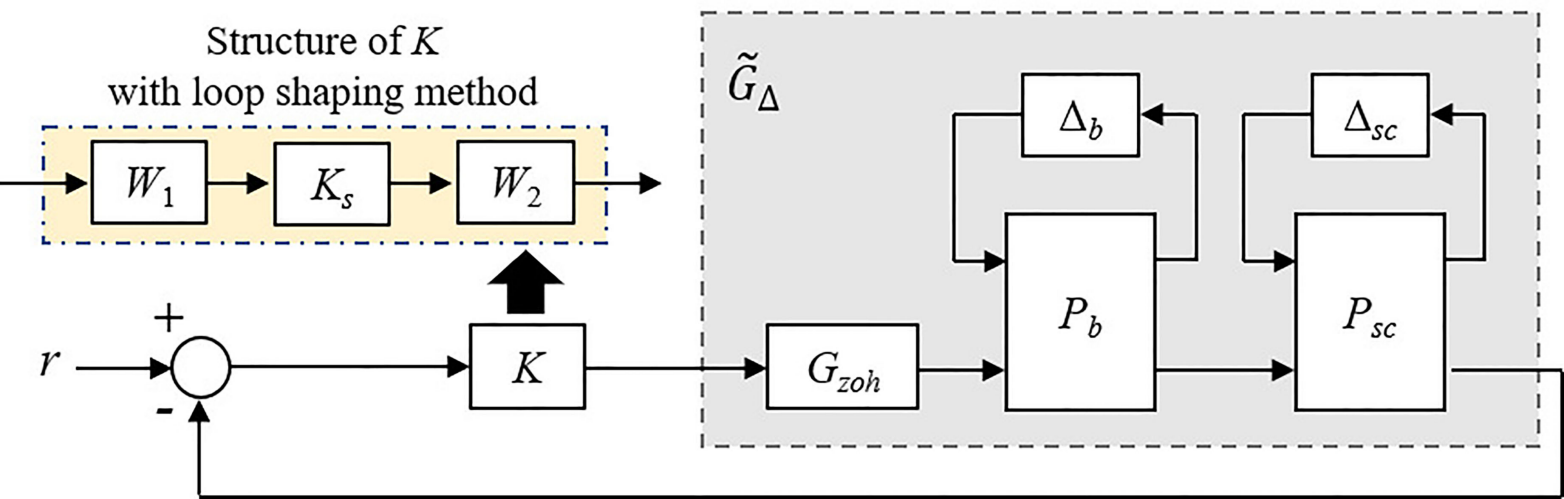
Feedback control setup for design purpose.

We then aimed to design a unique controller *K*(*s*) for a population of T1D patients – the adult cohort in this study case – able to maintain the BG level in a specified range despite T1D patient variabilities. For feedback controller design purpose, it is proposed to work on the feedback architecture given in **Figure 7**. The block *G_zoh_* (*s*) has been introduced to model the digital-analogue converter integrated in the insulin pump, as a delay of *T_s_*/2 where *T_s_* is the considered sample time. Here, we modelled *G_zoh_* (*s*) by a Pade approximation of first order. Hence, the unique controller *K*(*s*) must be designed to control the augmented system ${\tilde{G}}_{\Delta }(s)$ shown in **Figure 7**. In this work, the loop shaping method fitted with an *H_∞_* optimization problem was used to guarantee robustness and the closed-loop stability (40). Such robust technique usually involves two main steps, *i*) define a pre-compensator *W*_1_(*s*) and a post-compensator *W*_2_(*s*) to enforce the desired open-loop specifications on the shaped plant ${\tilde{G}}_{s}(s)=W_{2}(s){\tilde{G}}_{\Delta }(s)W_{1}(s)$ and *ii*) use the normalized coprime factor (41) to solve an *H*_∞_ optimization problem according to (40). All theoretical justifications dedicated to the considered Glover-McFarlane *H*_∞_ normalized coprime factor loop-shaping algorithm are given in (40, 41).

According to (40), we consider the nominal plant ${\tilde{G}}_{0}(s)$ (Δ*_sc_* = 0 and Δ*_b_* = 0) for design purpose. Thereby, the constructive solution based on equation (11) becomes a crucial step to obtain the smallest conservative LFT. To design a controller *K_s_* (*s*) able to stabilize a family of systems of the nominal shaped plant ${\tilde{G}}_{s0}(s)=W_{2}(s){\tilde{G}}_{0}(s)W_{1}(s)$ , weighting functions *W*_1_(*s*) and *W*_2_(*s*) have to be defined. In a preliminary study (28), PID controllers achieved acceptable performance and the worst-case performance was observed for the patient 8 of the adult cohort. Thus, we selected *W*_2_ = 1 and chose the continuous state-space representation of the individualised Proportional-Integral-Derivative (PID) controller dedicated to the eighth T1D patient for *W*_1_(*s*). Interested reader can refer to (29) to have the guidelines for PID tuning with two physiological parameters: the body weight and the total daily insulin dose. The last optimization step can be applied to improve worst-case results and be robust against the uncertainty ball in the normalized coprime factors. With the following *H*_∞_ cost function:


(13) $$\gamma (K_{s}(s))={\left\|\left[\begin{array}{c} 1\\ K_{s}(s) \end{array}\right]\left(1-{\tilde{G}}_{s0}(s)K_{s}(s)\right)[1\;{\tilde{G}}_{s0}(s)]\right\|}_{\infty }$$

the optimal performance is obtained by minimizing the following cost:


(14) $$\gamma :=\underset{K_{s}}{\min}\gamma (K_{s}(s))$$

γ is linked with the normalized coprime stability margin. In the range 1<γ<3, stability margins are judged satisfactory to be robust against the considered unstructured uncertainties. In our case, we are in this expected range (γ = 1.69). Hence, the unique robust feedback controller *K*(*s*) for a population of T1D patients is finally built by combining the *H*_∞_ controller *K_s_* (*s*) designed on the worst-case results, with the shaping functions *W*_1_(*s*) and *W*_2_(*s*) according to *K*(*s*) = *W*_1_(*s*)*K_s_* (*s*)*W*_2_(*s*).

Note that the authors are aware that a *µ*-analysis should be required to know if the resulting controller is able to theoretically satisfy the control specifications for all uncertainties Δ*_sc_* and Δ*_b_*. Due to the scope of the journal, it is proposed here to only perform several simulations in the result section to assess this requirement. Interested readers can however consider the preliminary works (28, 31) to know how this concern can be theoretically addressed.

### Metrics for Closed-Loop Therapy Assessment

In this study, eight of the metrics recommended in (42–44), *i.e.* the Time Below Range (TBR) with too different levels, the Time In Range (TIR), the Time Above Range (TAR) with also two different levels, the Low Blood Glucose Index (LBGI), the High Blood Glucose Index (HBGI), and the mean BG, were used for performance assessment. In addition, we also considered the Total Daily Insulin (TDI). For the time spent in the different glycaemic ranges the targets recommended in (45) for normal T1D adult patients were used. Definitions of these metrics and recommended targets are provided as **Supplementary Material**. Note that there is no official recommended value or target for the TDI metric. Indeed, the insulin need is highly dependent on the physiological status (e.g., stress, physical activity) and characteristics of the patients. This metric was therefore used to monitor the aggressiveness of the studied closed-loop solutions, and for comparison purpose.

### Statistical Analysis

To complete the performance analysis, normality of datasets was tested using the Shapiro-Wilk test and statistical significance was then assessed using either the two-sided paired sample t-test or the two-sided Wilcoxon signed rank test. P-values lower than 0.01 were considered significant.

## Results

As mentioned above, the objective of this work is to present and assess two different manners to handle the interpatient variability, which still challenges AP systems. The first subsection presents the results of a highly individualised approach with the islet-based closed-loop (**Figure 3**). In contrast, the second subsection presents the results obtained with a more common CGM-based AP system where a unique controller is tuned, for the whole adult cohort of the T1DMS, using a robust control theory approach (**Figure 5**). The results presented in both subsections are based on meal scenarios individualised with the method presented in subsection 2.2. Two realistic 48-hour scenarios, where the same meal pattern is repeated on two consecutive days, are simulated. The first pattern, referred to as the “standard scenario”, consists in five carbohydrate intakes, 0.62, 0.96, 0.27, 1.10 and 0.27 grams of glucose per kilogram of body weight respectively at time *t* = 180, 480, 720, 900, and 1080 minutes (corresponding, on average, to 45, 70, 20, 80 and 20 grams of glucose over the whole adult population, see section 2.2). The second pattern, referred to as the “challenging scenario”, consists in three large carbohydrate intakes, 0.89, 1.24, and 1.10 grams per kilogram of body weight respectively at time *t* = 180, 480, and 960 minutes (corresponding to 65, 90 and 80 grams on average). The default meal duration of 15 minutes was used for all meals.

### Islet-Based Closed-Loop Therapy Assessment

**Figure 8:**
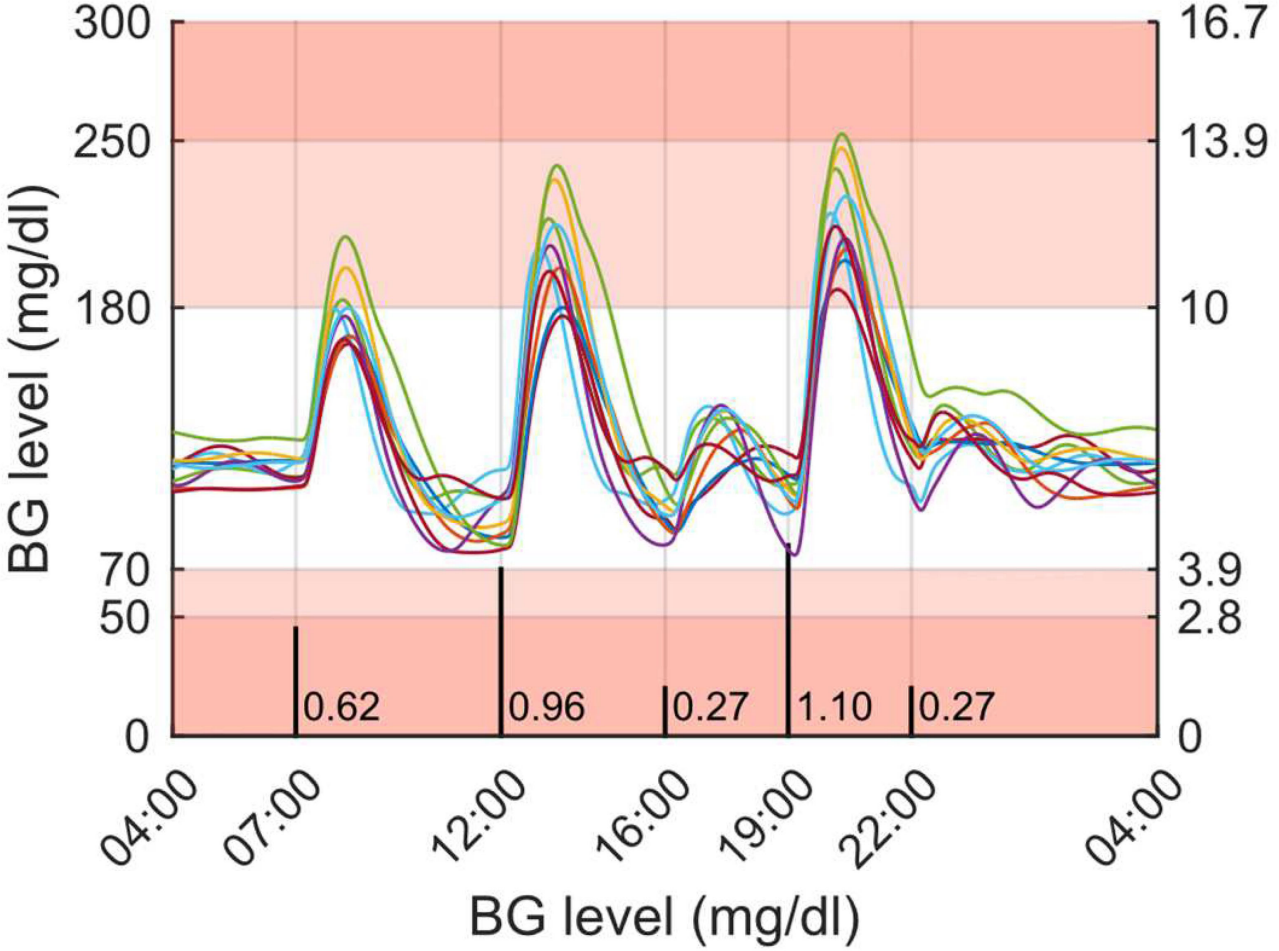
Simulation results for the ten T1D adults submitted to a 48-hour, 3-meal 2-snack scenario (last 24 hours are displayed) and treated with the islet-based closed-loop therapy (*via* subcutaneous routes). Meal intakes are labelled in g/kg and marked with black vertical bars on the chart. Regions with no glycemic risk, moderate glycemic risk and high glycemic risk are color-coded, respectively in white, pink and red.

**Figure 9:**
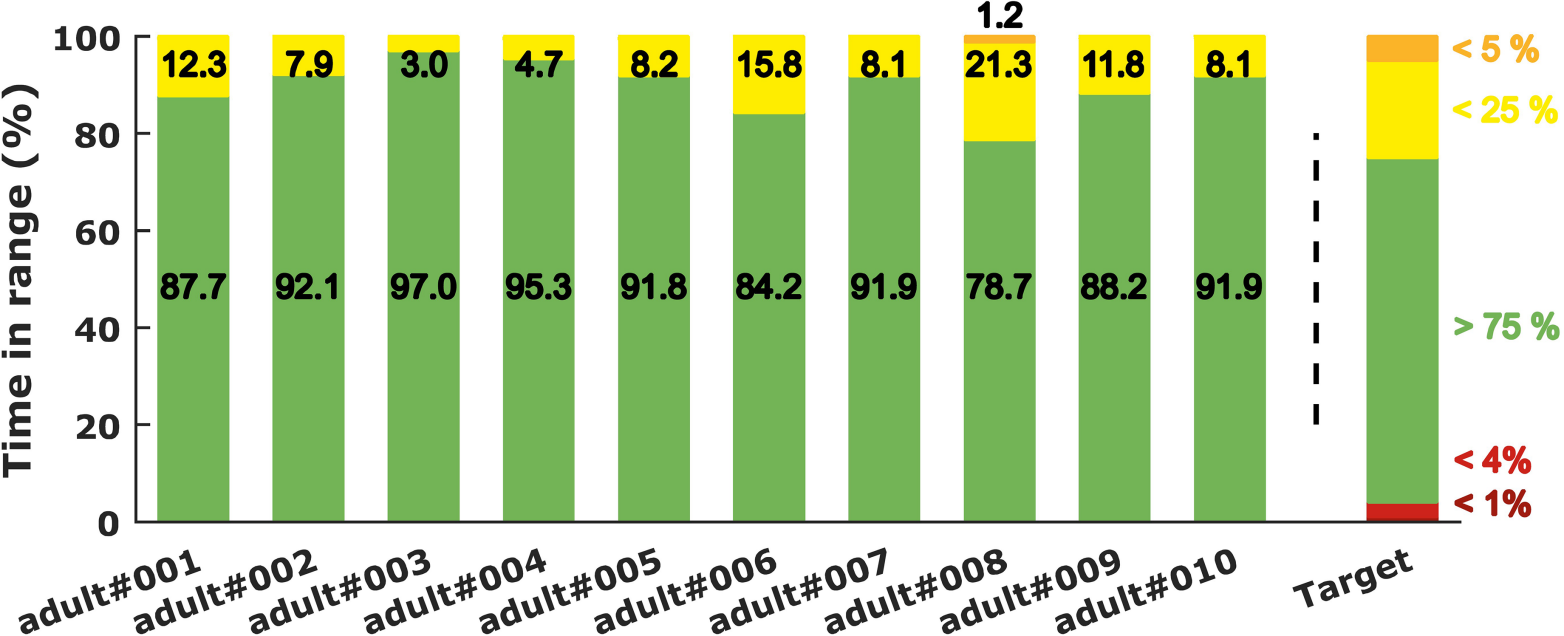
Time spent in the different glycemic ranges for the ten adults of the T1DMS treated with the islet-based BG regulation closed loop and submitted to the “standard scenario”. The recommended targets [see Battelino et al. (42)] are plotted on the right side of the chart.

Our GA-based tuning method was used to tune the parameters of a PD_BASAL_ controller for each adult patient of the T1DMS cohort. Contrary to our previously published works, the controllers are tuned here using individualised single meal scenarios (see Methods section). These controllers are associated with the biosensor model presented in (13) to form the islet-based closed loop. To assess the performance of this system, the ten T1D adults were submitted to the “standard scenario”. **Figure 8** presents the BG profiles obtained for each patient during the last 24 hours of this realistic 3-meal 2-snack scenario. For every patient, the BG regulation system provided satisfactory performance with limited postprandial hyperglycaemia and no hypoglycaemic event during the 48 hours. To complete the assessment of our islet-based closed-loop system, we computed the performance metrics detailed in the Methods section. Concerning the time spent in the 5 glycaemic ranges defined by Danne and colleagues (44), the islet-based closed loop permitted to all patients to reach the recommended targets (45). Excellent results were obtained for all the patients (TIR ranging from 78.7% to 97.0%) with a particularly satisfactory mitigation of the hypoglycaemic risk (TBR = 0% for every patient), see **Figure 9**. According to the T1DMS User Manual, the mean LBGI and HBGI are minimal for all patients (see **Table 1**). Concerning the mean BG, most patients present levels below 140 mg/dl, which allow them to achieve the recommended HbA1c^3^ target level of 6.5%^4^.

**Table 1:** Performance metrics for the 10 T1D adults treated with the islet-based closed loop and submitted to the “standard scenario”.

Patient	LBGI (-)	HBGI (-)	TDI (U)	Mean BG (mg/dl)
1	0.1	2.3	42.4	131.4
2	0.2	1.6	44.9	124.4
3	0.5	1.2	55.3	122.3
4	0.3	1.5	33.3	125.5
5	0.4	1.6	39.1	123.3
6	0.2	2.8	68.3	134.7
7	0.6	1.6	39.5	120.4
8	0.2	4.1	59.7	146.6
9	0.1	2.4	32.0	132.4
10	0.1	1.6	44.8	125.2

The metrics extracted for this analysis are the Low Blood Glucose Index (LBGI) (unitless), the High Blood Glucose Index (HBGI) (unitless), the Total Daily Insulin (TDI) units injected, and the mean BG level (Mean BG).

### Assessment of the Robust Closed-Loop Therapy

We integrated the unique robust controller *K* and the bolus calculator rule (4) (both described in the Methods section) in the UVA/Padova T1DMS according to the setup shown in **Figure 5**. Closed-loop was assessed with respect to standard recommendations introduced previously. A standard CGM sensor model was used for these simulations. Ten T1D adults were submitted to two multi-meal scenarios: the first one is the “standard scenario” mentioned above (a 3-meal 2-snack pattern repeated on two consecutive days) and the second is the “challenging scenario” (three heavy meals daily for 48 hours). Scenarios were also individualised using the patient body weight. As for the islet-based closed-loop, the performance assessment was made on the last 24 hours (second day). In addition, scenarios were repeated 25 times to account for the random inaccuracies of the CGM sensor (46).

**Figure 10:**
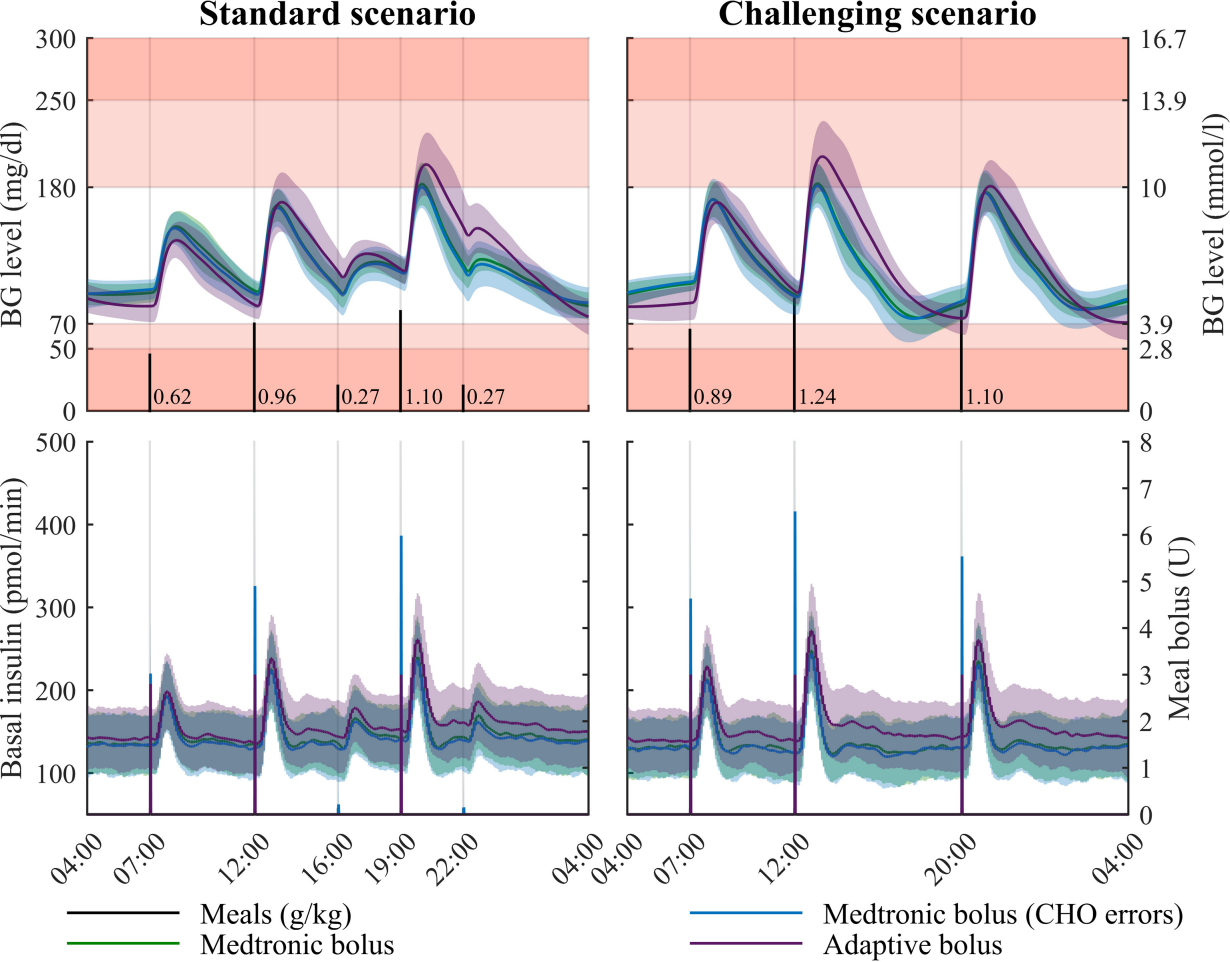
Simulation results for two realistic 48-hour multi-meal scenarios in adults (last 24 hours are displayed), for three closed-loop therapies. The proposed robust controller is assessed with three different meal bolus solutions: the Medtronic bolus, the Medtronic bolus with errors introduced in the patient-provided carbohydrate (CHO) counting, and the proposed adaptive bolus. Mean glucose profile (curve) and standard deviation (coloured patches) are displayed on the top panels. Regions with no glycaemic risk, moderate glycaemic risk and high glycaemic risk are color-coded, respectively in white, pink and red. Bottom panels display the basal and bolus insulin infusion for the three evaluated closed-loop (left axis for the basal and right axis for the bolus).

For both scenarios, three closed-loop therapies were evaluated. In all cases, the unique robust controller designed in subsection 2.3 was used. The changes only concern the insulin bolus delivery triggered by the announcement of a sizeable meal. First, we considered the standard of Medtronic’s bolus rule [see for instance the equation (6) of (46)] with a perfect estimate of carbohydrate (CHO) intakes, *i.e*. the patient enters, to the nearest gram, the exact carbohydrate content of the meal into the AP device. Since it has been reported that errors in carbohydrate counting by patients can range from -30% to +40% (47), a second series of simulations is performed with the same therapy, but with random CHO estimation errors. Finally, the third closed-loop therapy evaluated here, integrates the proposed meal bolus solution called “adaptive bolus”, and whose bolus rule is given in the equation (4). **Figure 10** and **Table 2** show the simulation results and the corresponding performance metrics.

**Table 2:** Metrics for closed-loop therapy assessment - Robust control laws - Adult cohort.

Standard scenario									
Category	TBR 2 (%)	TBR 1 (%)	TIR (%)	TAR 1 (%)	TAR 2 (%)	LBGI (.)	HBGI (.)	Mean BG (mg/dl)	TDI (U)
MED	0.0 (0.1)	0.5 (1.6)	97.1 (3.5)	2.4 (3.2)	0.0 (0.0)	0.6 (0.4)	1.0 (0.5)	117.9 (5.6)	48.4 (12.3)
MED-ERR	0.5 (2.1)	1.8 (4.0)	95.8 (5.6)	2.4 (3.3)	0.0 (0.0)	0.9 (1.0)*	1.0 (0.5)	116.3 (6.5)*	48.8 (12.8)*
ADAPT	0.2 (0.8)	3.0 (4.4)	90.6 (10.1)	6.4 (7.6)	0.6 (1.8)	1.1 (0.7)*	1.7 (1.3)	121.5 (9.8)	46.8 (10.7)*†
Challenging scenario									
Category	TBR 2 (%)	TBR 1 (%)	TIR (%)	TAR 1 (%)	TAR 2 (%)	LBGI (.)	HBGI (.)	Mean BG (mg/dl)	TDI (U)
MED	0.5 (1.7)	4.6 (6.4)	90.5 (11.3)	4.8 (6.3)	0.0 (0.0)	1.6 (0.9)	1.3 (0.6)	113.7 (3.5)	48.9 (12.7)
MED-ERR	1.9 (4.1)	8.0 (7.5)*	86.9 (10.8)	5.0 (5.7)	0.0 (0.0)	2.2 (1.7)*	1.3 (0.6)	112.6 (5.8)	49.5 (13.1)*
APAPT	1.1 (3.0)	6.5 (8.9)	84.1 (16.4)*	9.4 (9.5)*†	0.8 (2.4)	2.0 (1.3)*	2.2 (1.5)	119.2 (8.5)*†	46.6 (10.7)*†

Simulation results were obtained with the 10 adult patients of the T1DMS. Three closed-loop strategies are compared: the unique robust controller K fitted with the bolus calculator of Medtronic without (MED) and with CHO counting errors (MED-ERR), and the proposed meal-independent adaptive bolus rule associated to the unique robust controller K (ADAPT) shown in **Figure 5**. Standard and challenging individualised meal scenarios consider realistic daily glucose intakes of a five-meal intakes (45g, 70g, 20g, 80g and 20g) and three heavy meals (65g, 90g, 80g). The metrics extracted for this comparison are the Time Below Range (TBR) (level 1 and 2), Time In Range (TIR), Time Above Range (TAR) (level 1 and 2), the Low- and High- Blood Glucose Index (LBGI and HBGI), mean Blood Glucose (Mean BG) concentration in mg/dl, and the Total Daily Insulin (TDI) in units of insulin. Standard Deviations (SD) are displayed for all metrics, see values into the parentheses. Symbol * indicates statistical significance (p<0.01) with respect to MED and symbol † indicates statistical significance (p<0.01) with respect to MED-ERR.

For the “standard scenario” (left plots of **Figure 10**), the BG levels remained mostly in the TIR interval without snack bolus. The same trend occurred for the “challenging scenario” reported on the right side of **Figure 10**. For the so-called “standard scenario”, the three assessed closed-loop insulin therapies presented a mean TIR above 90% (see **Table 2**). Moreover, the unique controller fitted with the adaptive meal-independent bolus rule possessed the smallest TDI, causing *de facto* a small increase of TAR1, TAR2 and HBGI metrics. Nevertheless, all metrics followed the recommended values, see **Supplementary Material**. These data motivated the use of a unique robust controller designed according to the protocol introduced in section 2.3. As one would expect, the best TIR (97.1%) was obtained with the standard rule of Medtronic bolus delivery, without CHO errors. Removing the CHO estimation in the bolus calculator (*i.e*., using the adaptive bolus) caused a performance drop of 6.5% for the TIR. An equivalent gap (6.4%) can be observed with the second scenario. However, this gap was attenuated when the CHO counting errors were considered: the drop decreased to 5.2% between the Medtronic bolus with CHO errors and the adaptive one in the standard scenario, and was further reduced to 2.8% in the challenging one. In other words, when realistic CHO counting is considered, the price to pay to mitigate the patient’s workload and anxiety is a deterioration of the time spent in the TIR of 2.8% on the last 24 hours, with slightly better results for the TBR2, TBR1 and LBGI metrics.

### Comparison With Other Works

During the last decade, many algorithms have been proposed for the Artificial Pancreas controller and tested with the UVA/Padova T1DMS (48–51). Varying levels of closed-loop performance have been achieved *in silico* depending on the complexity of the control algorithm and on the degree of user input (meal and exercise announcement). To compare our results to the literature, we selected recent works, published by Gondhalekar et al. (50) and Colmegna et al. (51), for their similarities with our work and their use of the same simulator version, which enables a fair comparison. The meal scenarios used in these works were simulated 25 times for each adult of the T1DMS cohort (note that the individualisation function presented in section 2.2 was not used here). In (50), a MPC law which uses a discrete-time LTI model of glucose-insulin dynamics is proposed. The control algorithm integrates two main features: a velocity-weighting to mitigate controller-induced hypoglycaemia and a velocity-penalty to correct postprandial hyperglycaemia. For this comparison, our unique controller *K* was associated with the Medtronic bolus rule. The 27-h meal scenario consisted in three large glucose intakes of 90 grams each. Contrasting results were obtained as the TIR increased by 8.8% with our controller but with a poorer mitigation of the hypoglycaemic risk (2.53% vs 0.07% - see **Table 3**). Of note, this comparison presents two limitations lying in the number of patients used [the full cohort of 111 patients was used in (50)] and the different premeal bolus strategies. In (51), Colmegna and colleagues proposes a control strategy based on hyperglycaemia detection to switch between two controllers of varying aggressiveness, both designed using an LPV model of the glucose-insulin dynamics and the *H*_∞_ framework. This second comparison permitted to assess the performance of our unique robust controller alone, *i.e*., without premeal bolus. The 28-h meal scenario consisted in 3 glucose intakes of 40, 70 and 60 grams of glucose. Here, our controller was outperformed by the switching controller which permitted both a lower TBR (respectively 2.96% vs 0.00%) and a better TIR (88.0% vs 73.4%). This result did not come as a surprise since the design of a unique controller for the whole adult cohort (our work), compared to two individualised controllers per patient in (51), induces reduced closed-loop performance due to an increased conservatism.

**Table 3:** Performance indicators for the comparison with literature.

Ref.	Controller	Premeal Bolus	Mean BG [mg/dl]	TBR 2 [%]	TBR 1 [%]	TIR [%]	TAR 1 [%]	TAR 2 [%]	Risk index	
									LBGI	HBGI
This work^a^	*H*_∞_	Yes	119.7	0.09	2.53	88.1	9.33	0.00	1.11	1.77
(50)^b^	MPC	Yes	N/A	0.00	0.07	80.8	19.2	1.80	0.12	3.63
This work^a^	*H*_∞_	No	145.2	1.33	2.96	73.4	23.7	2.91	1.00	4.59
(51)^a^	Switched *H*_∞_	No	133.2	0.00	0.00	88.0	12.0	N/A	0.32	2.49

^a^Simulated in T1DMS S2013 with 10 adult patients.

^b^Simulated in T1DMS S2013 with 111 adult patients. N/A, not applicable.

## Discussion

### Modelling the Biological Diversity to Improve Simulation Realism

Variability in diabetes takes many forms, which can be classified as inter- and intra-patient variabilities. Intra-patient variability is linked to the evolution, over time, of the general body status and physiological features for each T1D patient. Interpatient variability corresponds to the variation of body characteristics between patients, by genetic differences and environmental factors, since past and present lifestyles shape the body and its response to nutrient intake. These variabilities result in a very specific response to meal intake which, paired with the individual response to insulin therapy, still constitute major hurdles to the development of fully automated Artificial Pancreas systems able to truly restore glucose homeostasis. In this context, numerical simulation tools are now commonly used to assess control algorithms with respect to different sources of variability in a cost-effective manner. In particular, the UVA/Padova T1DM Simulator accurately models the interpatient variability observed in response to meal intake in real T1D patients (52).

Concerning the intrapatient variability, we are aware that one limitation of the version 3.2 of the UVA/Padova T1DMS (the version used in this work), is the time-invariant definition of some important physiological parameters, e.g., Insulin Sensitivity (IS), which has been clearly stated in the Assumption 1. This limitation led the US FDA to approve the simulator for single-meal simulations only (21). Several methods were proposed to address this issue (53–55). In particular, Visentin et al. proposed in (55) an upgrade of the T1DMS where a time-varying definition of the model parameters *k_p_*_3_ and *V_mx_* is used to account for the intraday and interpatient variabilities of IS. This version of the T1DMS is however not commercially available yet.

In the context of the DIABLO project, other limitations of the T1DMS were highlighted in a precedent *in silico* work, where the simulator was used to validate the concept of an islet-based closed-loop therapy (13). The current metabolic model of the UVA/Padova T1DMS cannot model the dynamics of lipids, amino acids and hormone concentrations in blood, besides insulin and glucagon, which all reflect the general body status. As we already demonstrated *in vitro* that our biosensor properly captures the modulation of islet responses induced by GLP-1, adrenaline, and amino acids (11, 14), it is impossible to fully assess *in silico* the potential of our biosensor with the current metabolic model of the T1DMS. The secretion of GLP-1 by intestinal cells is closely related to nutrient intake (56). GLP-1 concentration variations could thus be extrapolated from variables already modelled in the simulator, e.g., glucose mass in intestine, rate constant of intestinal absorption. However, it appears more complicated to include adrenaline, fatty acids or amino acids concentration variations to the T1DMS metabolic model without new clinical data. Despite the above-mentioned limitations, the version 3.2 of the UVA/Padova T1DMS still is a powerful tool to assess different approaches to handle interpatient variability and compare control strategies towards the integration of our islet-based biosensor in an AP system.

### Result Analysis and Learnt Lessons for Interpatient Variability Management

**Figure 11:**
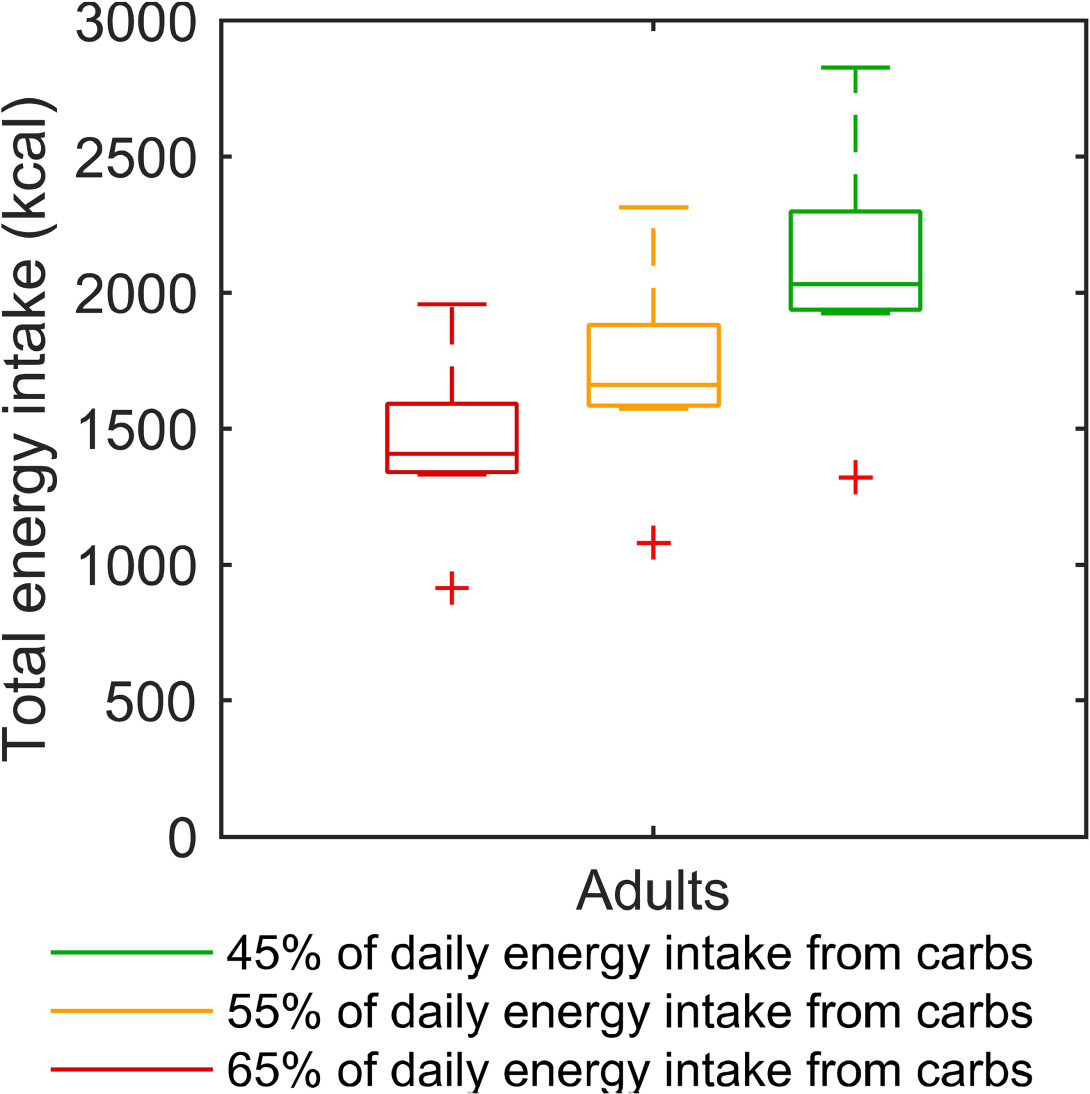
Boxplots of the daily energy intake corresponding to the individualised “standard scenario” of each virtual adult. Three hypotheses are considered for the proportion of energy intake covered by carbohydrates: 45%, 55%, and 65%. For each box, the central mark and the edges of the box are respectively the median and the 25th and 75th percentiles. Data points, without outliers, are delimited by the whiskers, and outliers are plotted individually as red crosses.

To elaborate further on the modelling of interpatient variability, we developed a method which accounts for the specific energy need of each T1D patient by individualising meal scenarios based on patient body weight. Our meal scenario individualisation method is not a built-in feature of the T1DMS, and therefore needs to be discussed further. To ascertain that the method yields realistic glucose intake distributions for T1D adults, we computed the daily energy intakes corresponding to the daily glucose intakes outputted by the individualisation function. We considered three hypotheses regarding the proportion of daily energy intake provided by carbohydrates: 45%, 55% and 65%. These hypotheses are in line with the American Diabetes Association recommendation for T1D adults: 45-60% of energy requirements covered by carbohydrates (57). The corresponding daily energy intakes are plotted in **Figure 11** for each hypothesis. Unsurprisingly, the total energy intake increased when the proportion of carbohydrates decreased, and fell between 1300 to 2800 kcal/day depending on the carbohydrate proportion hypothesis. As this result is consistent with the range of daily energy intakes reported in the literature for T1D adults (34, 58), we can conclude that the weight-dependent definition of meal scenarios is functional for adult patients, and does not yield aberrant results.

Furthermore, the benefits of numerical simulation were exploited to assess two different approaches to handle interpatient variability. First, controllers highly individualised using our GA-based optimisation method and individualised meal scenarios were used to define the best performance that could be achieved with a biosensor-based closed loop and unannounced meals. In so doing, we intended to investigate the relative contribution of controller individualisation and control algorithm complexity. The results obtained with these highly individualised controller parameters were satisfactory as excellent regulation performance was observed without meal announcement. Compared to our previous work (13), the weight-dependant definition of our “standard scenario” resulted in a TIR improved by 1.0% on average (88.1% in (13) vs 89.1% here) with a similar standard deviation (5.4% vs 5.0%), thanks to a better mitigation of the hypoglycaemic risk, - 1.3% on average (1.6% vs 0.3%). In both cases, the adult cohort was submitted to a very similar 3-meal 2-snack scenario with an average daily glucose intake of 235 grams. As there is no other obvious reason why our controller tuning methodology would yield better performing controllers in the second case, we conclude that the use of a unique scenario for all patients could introduce a bias when assessing closed-loop systems with the T1DMS.

As the level of individualisation obtained with the GA could not realistically be achieved *in vivo*, controllers need to be tuned more conservatively. Through the DIABLO project, we thus investigated a second approach based on the design of a unique *H*_∞_ robust controller tuned for all virtual T1D adults and the infusion of a bolus to reduce postprandial hyperglycaemia. Note that we firstly developed this approach with a traditional CGM sensor. The advantage of this approach is that it could theoretically handle both intra- and interpatient variabilities. To manage trade-offs of control requirements, *H*_∞_ control theory is known as a powerful tool. Among the pioneering works, Kienitz et al. (59) addressed for the first time the BG regulation with *H*_∞_ control theory to manage the considerable amount of model uncertainty. This work has been followed by (60) where a sensitivity analysis provides the three-parameter set having the most significant effect on insulin and glucose dynamics. In spite of advances in the *H*_∞_ framework, it is important to underline that an efficient robust control solution can be obtained if and only if it is designed on an accurate model able to capture all variabilities. In this context, the work reported in (23) highlights the benefits of simulation by providing control-relevant nonparametric models identified from the UVA/Padova simulator. Based on the structure of LTI models, the authors proposed to model glucose-insulin dynamics by a unique LTI model of third order. This work encouraged us to develop the methodology introduced in section 2.3 where a family of thirteen-order linear models is derived by using mathematical formalisms like the unstructured multiplicative uncertainty and the LFT representation. From the results obtained with the proposed closed-loop architecture (**Figure 5**), acceptable performances (TIR above 90%) were reached thanks to the announcement of sizeable meals. The price to pay to be robust (or as insensitive as possible) against variabilities within a population of T1D patients with a standard CGM sensor (the default sensor configuration of T1DMS (21), with a sampling time of 5 min), is to include the patient in the loop.

To better assess the performance of our unique controller, we compared its performance to two control laws published recently: the velocity-weighting and velocity-penalty MPC law proposed by Gondhalekar et al. (50) and the switching LPV approach proposed by Colmegna et al. (51). The first conclusion of these comparisons introduced in section 3.3 is that safety features to prevent hypoglycaemia are necessary (an Insulin-On-Board (IOB) limitation in the control algorithm is implemented in both cases), even when meal announcement lessen the constraint on the controller. The second conclusion is that the need to include the patient in the loop could be relaxed by individualising the *H*_∞_ controller using the patient’s previous therapy parameters (e.g., the Total Daily Insulin as in (24)). In addition, control-oriented models have to be designed to capture other physiological factors than the ones included in the v3.2 of T1DMS. This statement motivated other investigators to develop LPV models capable to integrate the variability of insulin sensitivity in models used for control design purpose (27).

### Bridging Model-Based Control Theory and the Islet-Based Biosensor

As previously mentioned, the overall objective of the DIABLO project is to gather the sensing capabilities of pancreatic islets and the benefits of robust control theory in a biosensor-based AP system. Thanks to its sensitivity to other insulin secretion modulators, the biosensor could alleviate the patients’ burden by reducing the need for meal and physical activity announcement, while providing a new insight on the very specific response of each patient to nutrients. By providing a finer image of the patient’s physiological status and multiple signals, we hope that this sensor could also help solving the well-known problem of unstable diabetes. The different simulation campaigns presented in this paper allowed us to highlight the relative contribution of algorithms to the overall closed-loop performance of AP systems. From the first conclusion of the comparison study, hypoglycaemia-prevention features, such as IOB, seem to be necessary. In addition, modern hybrid closed-loop systems frequently integrate a hypoglycaemic alarm to trigger the suspension of basal insulin delivery, referred to as Low Glucose Suspend (LGS). Although the biosensor response presents a natural glucose-dependent hysteresis protecting from hypoglycaemia (11, 61), it may have a shortcoming that is worth mentioning: *β*-cell activity at low glucose, *i.e*., the SP frequency, is not yet fully explored and the biosensor output may eventually not suffice, when the patient BG level is below the islet glucose stimulation threshold, to trigger such hypoglycaemia-prevention feature. The co-integration of a CGM technology and our biosensing one into a single device, may thus be necessary. This proposal appears reasonable from a technological standpoint as a glucose-oxidase electrode could be placed on the same MEA as the pancreatic islets embedded in the biosensor, and meets the recommendation expressed in (2) to integrate new signals for algorithm improvement. The combined use of multiple input signals with an LPV formalism to capture other physiological factors (see the second conclusion of the comparative study), would also further highlight the benefits of *H*_∞_ robust control theory for the regulation of T1D patient’s BG level. In particular, this method could permit the development of a Multi-Input Single-Output (MISO) controller, involving a better dimensioning of the control problem and a possible improvement to manage variability.

**Figure 12:**
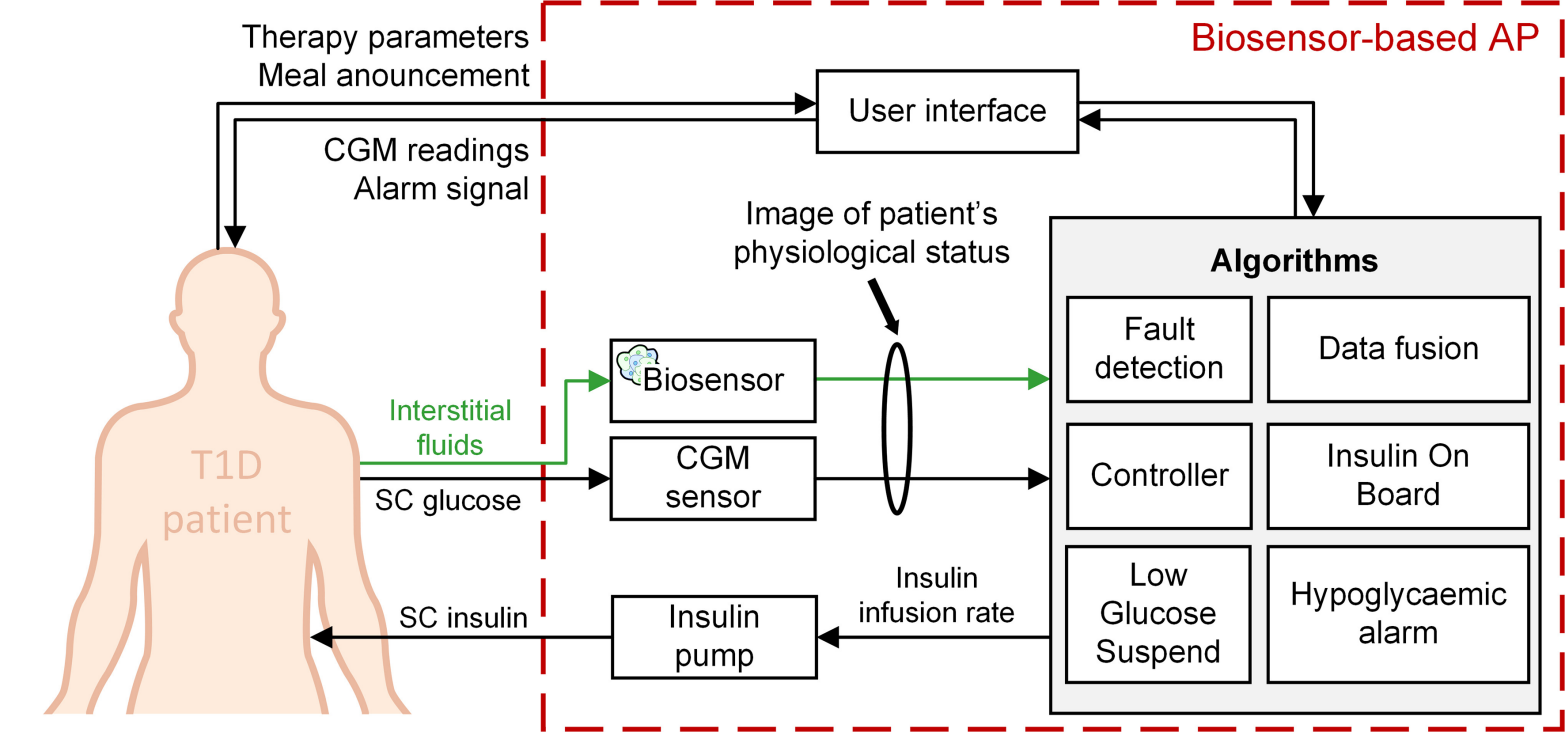
An Artificial Pancreas system based on two dissimilar sensors for insulin therapy of T1D patients.

At our current state of knowledge and advance of the biosensor, we thus propose the following setup for a realistic biosensor-based Artificial Pancreas (**Figure 12**). The integration of this two-sensor device could provide multiple signals to improve the performance of control algorithms (e.g., controller, bolus calculator, Insulin-On-Board estimation, fault detection). A data fusion algorithm could also be developed, to improve the real-time monitoring of patient’s physiological state, like in aeronautical systems (62). Such a system would be perfectly in line with the conclusions DCCT-EDIC study (63) concerning the need to mitigate hypoglycaemia in intensive insulin therapy and the recommendations formulated in (2) to integrate new signals to the Artificial Pancreas. Depending on our ongoing research, the set-up may in the long-term be simplified to a pure biosensor capable of detecting hypoglycaemic states by fully using the multiple inborn detection capacity of pancreatic islet sensors.

## Conclusion and Perspectives

Our previous work (13) presented an *in silico* proof-of-concept where a model of our biosensor was introduced in a closed-loop insulin delivery setup with PD_BASAL_ controllers individualised to meet patient’s specific insulin need and without meal announcement. In the current study, this control approach was assessed again, but with an important new aspect: the use of individualised meal scenarios. Concurrently, a model-based control approach was also introduced to tackle the variability observed within a real patient cohort with a unique robust controller and an adaptive meal announcement feature. As a first step, this approach was developed with a traditional CGM sensor and in a LTI context. The simulation results thereby obtained are discussed to highlight both the advantages and limitations of our biosensor, and the contrasted performance of our unique robust controller, which struggled to mitigate hypoglycaemia although providing satisfactory TIR for the whole adult T1D cohort. A conceptual work was finally conducted to sketch the outline of a realistic biosensor-based Artificial Pancreas where robust control theory could help to manage the integration of new signals. In line with the conclusions of this work, future investigations shall focus on the development of a MISO control algorithm, paired with LPV modelling of the glucose-insulin dynamics, and integrating hypoglycaemia-prevention features (e.g., IOB, LGS). The realism of simulations could be improved in future works *via* the introduction of additional variability sources (e.g., circadian insulin sensitivity variation, random meal time and content) to better model the real-life challenges of diabetes treatment, and enable the assessment of closed-loop therapies through multiple day/week simulation campaigns.

## Data Availability Statement

The raw data supporting the conclusions of this article will be made available by the authors, without undue reservation.

## Author Contributions

LO performed the simulation work, contributed to model development and wrote much of the manuscript. AP analysed the experimental data and developed the model. LC, RF, DG-D, HR, AFL, JC, and DH developed codes for robust control strategies in MATLAB. FL and MR provided the experimental data. SR, JL, DH, JC, AFL, and BC secured funding. All the authors contributed to the discussions. All authors contributed to the article and approved the submitted version.

## Funding

The authors wish to thank various Funding agencies. ANR (HyBiopacs to JL and SR, Isletchip to BC, JL, and SR, DIABLO to SR, JL, BC, and DH), Région d’Aquitaine (to BC, SR, and JL), French Ministery of Research (to MR). More precisely, this work has been supported by the French National Agency for Research (DIABLO ANR-18-CE17-0005-01), ECOSNord (M18M01) and SEP-CONACYT-ECOS-ANUIES under Grant 296692. This research has also been supported by the Fonds Européen de Développement Régional (FEDER) under the grant agreement DIAGLYC N°3538519.

## Conflict of Interest

The authors declare that the research was conducted in the absence of any commercial or financial relationships that could be construed as a potential conflict of interest.

## Publisher’s Note

All claims expressed in this article are solely those of the authors and do not necessarily represent those of their affiliated organizations, or those of the publisher, the editors and the reviewers. Any product that may be evaluated in this article, or claim that may be made by its manufacturer, is not guaranteed or endorsed by the publisher.

## Footnotes

1 GA, Genetic Algorithm.

2 CIR, Carbohydrate-to-Insulin Ratio.

3 HbA1c, glycosylated hemoglobin A1c.

4 The conversion was computed using the online eAG/A1C Conversion Calculator provided by the American Diabetes Association.
