# Supplementary material for: Towards the Integration of an Islet-Based Biosensor in Closed-Loop Therapies for Patients With Type 1 Diabetes
Source: Front Endocrinol (Lausanne). 2022 Apr 22;13:795225. doi: 10.3389/fendo.2022.795225 (PMC9072637; doi:10.3389/fendo.2022.795225)
Supplement: Supplementary file 1 [file DataSheet_1.pdf]

Supplementary Material

Metric definitions and their recommended values

Metrics	Definitions	Recommended values
TBR2 (%)	Percentage of the time spent below 54 mg/dl (3.0 mmol/dl) on 24h	TBR 2 < 1%
TBR1 (%)	Percentage of the time spent below 70 mg/dl (3.9 mmol/dl) on 24h	TBR 1 < 4%
TIR (%)	Percentage of the time spent in normoglycaemia (between 70 to 180 mg/dl) on 24h	TIR > 70%
TAR1 (%)	Percentage of the time spent above 180 mg/dl (10 mmol/dl) on 24h	TAR 1 < 25%
TAR2 (%)	Percentage of the time spent above 250 mg/dl (13.9 mmol/dl) on 24h	TAR 2 < 5%
LBGI (unitless)	This metric is derived from the Blood Glucose Index (20) and underlines the frequency of hypoglycaemic events. The specifications given in the T1DMS User Manual provide the classification given in the column entitled "recommended values".	LBGI is judged:<br>- minimal is lower than 1.1,<br>- low (1.1 ≤ LBGI < 2.5),<br>- moderate (2.5 ≤ LBGI < 5),<br>- high if higher than 5.
HBGI (unitless)	This metric is based on the blood glucose index (20) and underlines the frequency of hyperglycaemic events. The specifications given in the T1DMS User Manual provide the classification given in the column entitled "recommended values".	HBGI is judged:<br>- minimal is lower than 5,<br>- low (5 ≤ HBGI < 10),<br>- moderate (10 ≤ HBGI<15),<br>- high if higher than 15.
Mean BG (mg/dl)	It is a usual performance index in insulin therapies. This metric tends to replace the HbA1C measurement which is the "gold-standard" one	The closest to the desired target.
TDI (U)	Total insulin delivered to the patient over 24h.	No official recommendation.
